# Long-term effects of tocilizumab on retinal and choroidal inflammation in Birdshot uveitis

**DOI:** 10.1186/s12348-024-00443-9

**Published:** 2024-11-21

**Authors:** Lynn S. zur Bonsen, Daniel Schulze, Steffen E. Künzel, Anne Rübsam, Uwe Pleyer, Dominika Pohlmann

**Affiliations:** 1 Department of Ophthalmology, Charité – Universitätsmedizin Berlin, corporate member of Freie Universität Berlin and Humboldt-Universität zu Berlin, Augustenburger Platz 1, 13353 Berlin, Germany; 2 Institute of Biometry and Clinical Epidemiology, Charité – Universitätsmedizin Berlin, corporate member of Freie Universität Berlin and Humboldt-Universität zu Berlin, Charitéplatz 1, 10117 Berlin, Germany; 3 Berlin Institute of Health at Charité – Universitätsmedizin Berlin, Charitéplatz 1, 10117 Berlin, Germany

**Keywords:** Angiography, Birdshot uveitis, Choroidal inflammation, Indocyanine green, Interleukin-6, Tocilizumab, Uveitis, Vasculitis

## Abstract

**Background:**

Tocilizumab (TCZ), an interleukin-6 receptor antagonist, is approved for treating rheumatic diseases and has demonstrated efficacy in managing refractory non-infectious uveitis (NIU). This retrospective study aimed to investigate the long-term effects of TCZ on inflammation in the retinal and choroidal compartments in Birdshot NIU.

**Methods:**

Eight patients (16 eyes) received TCZ and were included in the analysis. The primary outcomes measured were inflammatory activity in the retina and choroid, assessed by fluorescein angiography (FA) and indocyanine green angiography (ICGA) using the Angiography Scoring for Uveitis Working Group at baseline, 6, 12, 24, and 36 months.

**Results:**

The mean follow-up time with TCZ treatment was 33 months. At baseline, the median FA score was 14 (quartiles: 10.25, 15.25), which significantly decreased over time (at 36 months: 8 (5.5, 11); *p* = 0.004). In contrast, the ICGA score significantly increased within the first year (median at baseline: 5 (4.75, 7.25); at 6 months: 7 (6, 9.25); at 12 months: 7 (6.5, 9.25); *p* = 0.002), but returned to baseline levels after two years (at 24 months: 5 (5, 6.5); at 36 months: 5.5 (4, 7.5)). Central retinal thickness (CRT) improved significantly after 6 months (median at baseline: 295 µm (275, 322); at 6 months: 275 µm (251, 308); *p* = 0.01).

**Conclusion:**

TCZ is effective in reducing retinal vasculitis and CRT in refractory Birdshot uveitis over time, but might be less effective in managing choroidal inflammation. Further studies are needed to determine the optimal treatment strategies for TCZ therapy in NIU.

## Introduction

Chronic uveitis, characterized by persistent intraocular inflammation, poses a therapeutic challenge. Uveitis accounts for 5–20% of preventable blindness in the developed world and up to 25% of cases in developing countries [1]. Non-infectious uveitis (NIU) is the predominant form, accounting for 67–90% of cases in developed countries [2, 3]. The global incidence and prevalence rates of NIU vary widely, and most epidemiological studies did not differentiate between infectious and non-infectious causes. One study conducted in the United States estimated the prevalence of NIU among adults to be 121 cases per 100,000.^2^ The main goal of managing NIU is to achieve a state of quiescence and prevent relapses of the disease. Birdshot uveitis (BU) is a rare form of non-infectious posterior uveitis characterized by chronic inflammation in both the choroid and retina. It is strongly associated with the presence of the HLA-A29 antigen. Macular edema (ME) is a common complication, occurring in approximately 80% of patients.

Corticosteroids serve as a first-line therapy for rapidly controlling acute inflammation in NIU patients. However, their long-term use is associated with various systemic and ocular side effects. Therefore, it becomes necessary to use steroid-sparing agents such as conventional disease-modifying antirheumatic drug (DMARD) or biologic agents. Adalimumab, a TNF-α inhibitor, is currently the only biological treatment approved by the Food and Drug Administration (FDA) and the European Medicine Agency (EMA) for non-infectious intermediate and posterior uveitis. It effectively reduces the risk of uveitis flares and preserves visual acuity (VA) [4, 5]. Nevertheless, some patients exhibit inadequate control of inflammation or exacerbations due to a loss of efficacy or the emergence of antidrug antibodies (ADA) against adalimumab. ADA occur in 2.7 to 35% of patients [4, 6].

Tocilizumab (TCZ), a neutralizing monoclonal antibody targeting interleukin-6 receptor, has emerged as a promising treatment option. The role of TCZ in addressing the interleukin-6-mediated inflammatory pathways has been well-established in the field of rheumatological diseases, including rheumatoid arthritis, juvenile idiopathic arthritis (JIA), giant cell arteritis or cytokine release syndrome [7, 8]. IL-6, a pleiotropic cytokine produced by macrophages, monocytes, and T-lymphocytes, is strongly upregulated during infection and inflammation. IL-6 promotes differentiation of CD4 + T-helper (Th) cells into Th17 cells, which are associated with immune-mediated diseases such as NIU [9]. IL-6 can also induce differentiation of CD8 + T-cells into cytotoxic T-cells [9]. Previous studies demonstrated elevated IL-6 concentrations in the vitreous fluid of chronic uveitis patients [10, 11]. By targeting IL-6 receptor, TCZ can reduce ocular inflammation, as described in several studies [12–15]. The randomized controlled trial STOP-Uveitis reported that TCZ significantly reduced vitreous haze (VH), central retinal thickness (CRT), and improved VA in patients with NIU [16].

While the efficacy of TCZ in treating NIU has been demonstrated, there is a paucity of long-term data specifically addressing its effects on retinal and choroidal inflammation in BU patients. Therefore, this retrospective study investigates the impact of TCZ therapy in a real-life setting using multimodal imaging techniques over an extended follow-up period. By analyzing the fluorescein angiography (FA) and indocyanine green angiography (ICGA) data, this study highlights the different effect on retinal and choroidal inflammation, respectively. Furthermore, evaluating long-term changes in CRT and VA provides significant insights into the overall efficacy of TCZ. Understanding underlying inflammatory pathways is essential to optimize treatment strategies in managing chronic refractory BU.

## Materials and methods

Patient data from medical records including angiographic images were analyzed to assess changes in inflammatory activity over a follow-up period of up to 36 months. This study was conducted in accordance with the Declaration of Helsinki. Ethical approval was obtained from the local ethics committee (EA2/066/19). Informed consent was obtained from all subjects involved in the study.

Eight patients (16 eyes) with refractory BU were included in the study and analyzed. All patients were switched to TCZ because of inflammatory activity despite immunosuppressive therapy, including adalimumab. Previous therapies included prednisolone, azathioprine, methotrexate, cyclosporine, mycophenolate mofetil, adalimumab, infliximab, intravitreal dexamethasone, and intravitreal fluocinolone acetonide. Unless otherwise indicated in Table 1, the aforementioned therapies were discontinued prior to the commencement of TCZ therapy due to insufficient efficacy or systemic intolerance. The interval between the last injection of intravitreal dexamethasone was a minimum of six months, while the interval between the last injection of intravitreal fluocinolone acetonide was a minimum of three years. An exclusion criterion for the following analysis was the administration of an intravitreal steroid implant. After approval due to off-label use by the health insurance company, TCZ (RoActemra; Hoffmann-La Roche, Switzerland) was administrated subcutaneously (162 mg/ml) every week. In the event of reduced efficacy, TCZ was temporarily switched to intravenous administration at a dose of 8 mg/kg body weight at 4-week intervals. All patients were tested for tuberculosis by QuantiFERON ®-TB-Gold and X-ray of the lung before initiation of TCZ therapy.

**Table 1:** Patient characteristics

Patient	Uveitis subtype	Onset of uveitis	Previous immuno- suppression*	Start of TCZ therapy	Additional immuno- suppression*	TCZ dosage	Side effects
1	BU	2014	1, 2, 3, 4, 5, 6, 8	2018	1	162 mg s.c. every week/ 3 × 8 mg/kg i.v. every month (2020)	no
2	BU	2015	1, 4, 6	2018	1, 3	162 mg s.c. every week	no
3	BU	2014	1, 2, 4, 5, 6, 7, 8	2018	3	162 mg s.c. every week	no
4	BU	2011	1, 2, 4, 5, 6, 7	2019		162 mg s.c. every week	no
5	BU	2016	1, 4, 6, 8	2019	1, 8 (OD 2019 & 2022), 9 (OS 2021)	162 mg s.c. every week	no
6	BU	2017	1, 4, 6, 8	2019	1	162 mg s.c. every week/ 3 × 8 mg/kg i.v. every month (2019)	no
7	BU	2013	1, 4, 5, 6, 8	2019		162 mg s.c. every week/ 18 × 8 mg/kg i.v. every month (2019–2022)	upper respiratory tract infections
8	BU	2014	1, 4, 6, 8, 9	2021		162 mg s.c. every week	exanthema

^*^ 1 Prednisolone < 10 mg; 2 Azathioprine; 3 Methotrexate; 4 Ciclosporin; 5 Mycophenolate mofetil; 6 Adalimumab; 7 Infliximab; 8 Dexamethasone intravitreal; 9 Fluocinolone acetonide intravitreal; *BU* Birdshot Uveitis, *TCZ* Tocilizumab, *OD* right eye, *OS* left eye, *s.c.* subcutaneous, *i.v.* intravenous

The primary outcomes measured in this study were changes in inflammatory activity in the retina and choroid compared to the baseline. Both parameters were assessed using FA as well as ICGA. Widefield FA and ICGA were performed simultaneously using Spectralis HRA + OCT (SPECTRALIS®; Heidelberg Engineering, Germany). For FA intravenous injection of 5 mL 10% sodium fluorescein (Fluorescein ALCON® 10%, Alcon Laboratories Belgium, Belgium) and for ICGA intravenous injection of 500 mg ICG (Cardiogreen; Peasel and Lorei, Germany) diluted in 7.5 mL of 0.9% salt solution were applied.

**Fig. 1:**
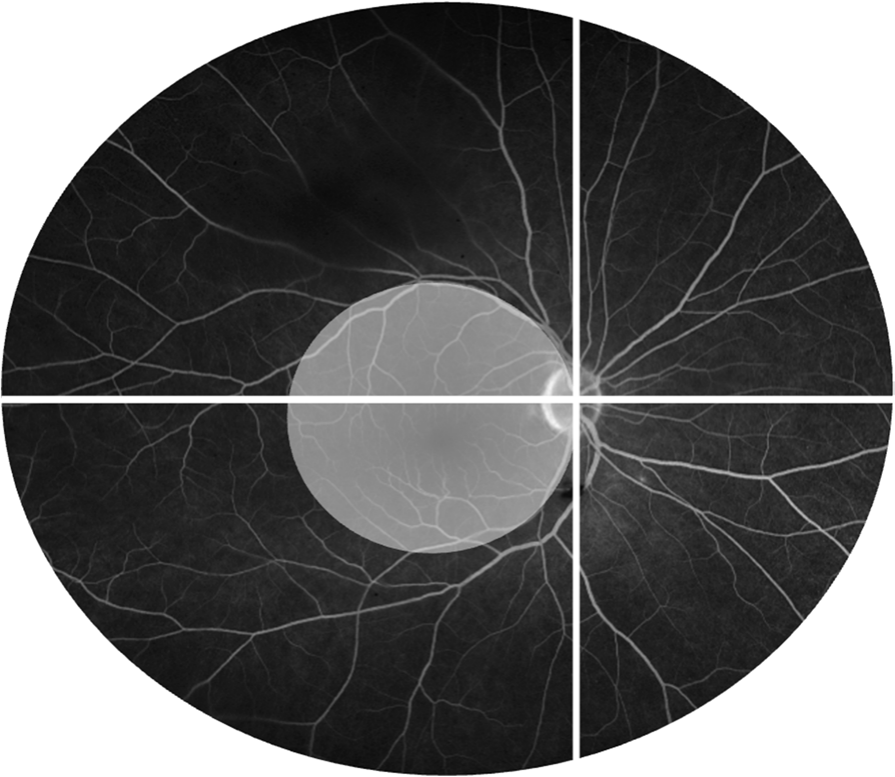
Example of a fluorescein angiography image showing the division into quadrants regarding the allocation of points in the angiography scores. The white circle illustrates the defined posterior pole [17]

**Fig. 2:**
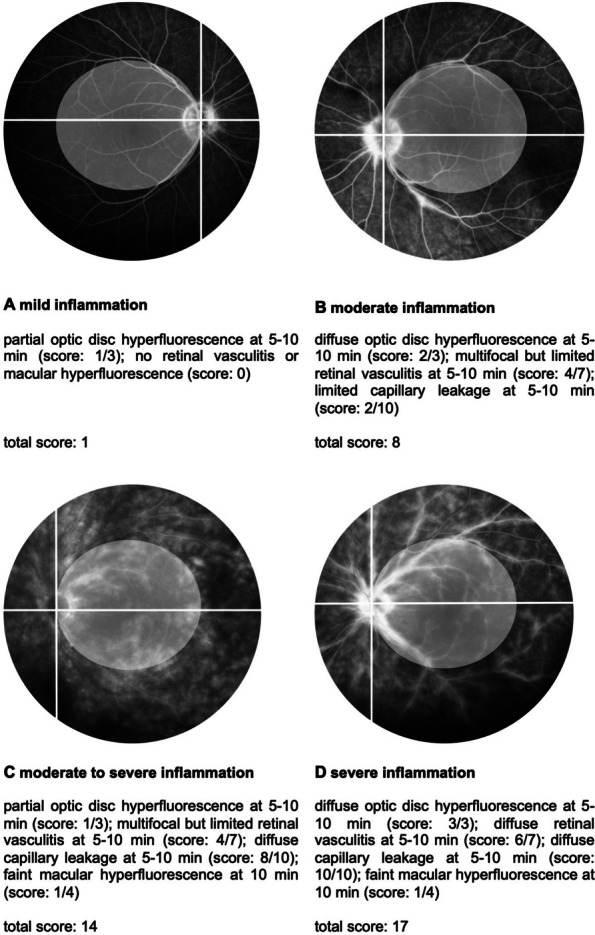
Fluorescein angiography score. For the complete calculation of the score, the points of the posterior pole, and of each quadrant were added together [17]

**Fig. 3:**
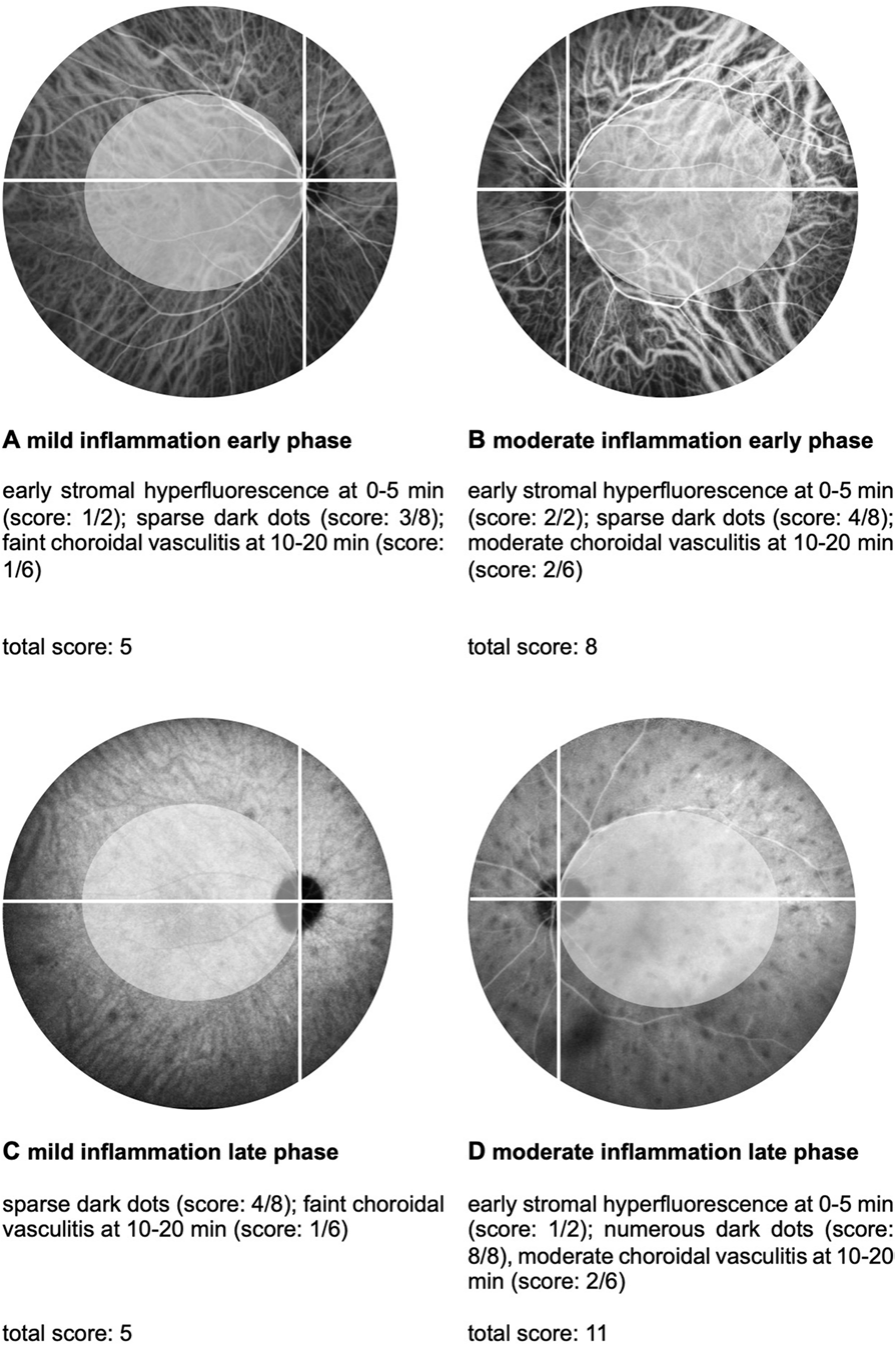
Indocyanine green angiography score. For the complete calculation of the score, the points of the posterior pole, and of each quadrant were added together [17]

The Angiography Scoring for Uveitis Working Group (ASUWOG) criteria were utilized to score and quantify the inflammation seen in the images [17]. Two graders evaluated the images independently. In cases of discrepancies, a third grader was consulted to reach a consensus. A score of up to 40 points can be assigned for inflammatory changes in FA. The score is assigned according to the extent of inflammation in each compartment such as optic disc hyperfluorescence, retinal and capillary leakage, or nonperfusion. For the graduation, the points will be counted in the corresponding categories per quadrant (Fig. 1). For ICGA respectively, the maximum score is 20 points. Scoring is based, for instance, on choroidal vasculitis or the presence of dark dots. Examples of the evaluation can be seen in Figs. 2 and 3.

The secondary outcome measures included a decrease in CRT, diminished VH score, and enhanced best corrected VA with TCZ therapy as compared to the initial measurements. CRT and the presence of ME was recorded using spectral domain optical coherence tomography (SD-OCT) (SPECTRALIS®; Heidelberg Engineering, Germany). ME was defined as CRT > 350 µm and/ or the presence of intraretinal or subretinal fluid in OCT B-scans. CRT was determined by the macula thickness map of Heidelberg Spectralis OCT. VH was classified through slit lamp examination according to the criteria by Standardization of Uveitis Nomenclature (SUN) Working Group [18]. Baseline measurements for all outcomes were taken at the initiation of TCZ therapy, with subsequent measurements recorded at 6, 12, 24, and 36 months.

De-identified patient data was collected from electronic medical record system and subsequently analyzed using R (version 4.3.0; [19]). Descriptive statistics are displayed as medians and quartiles. For depicting time courses, longitudinal linear mixed models were run that allowed describing individual trajectories as well as a general trend (linear random intercept random slope models with an auto-regressive-1 covariance structure). For the ordinal VH scoring (0, 0.5, ≥ 1), ordinal mixed models were used. Wilcoxon-tests were used to compare selected pairwise measurements for having an outlier-robust alternative for our small sample. P-values < 0.05 were considered as statistically significant and were interpreted in an exploratory manner.

## Results

The analysis involved 16 eyes of eight patients with refractory BU. At baseline, the mean age of the patients was 52.1 years (range: 35–69), with 50% being female. All patients were switched to TCZ because their inflammatory activity remained elevated despite treatment with different immunosuppression. Immediately prior to the initiation of TCZ therapy, the following medications were administered to patients but were discontinued due to insufficient therapeutic effects: one patient received cyclosporine A, one patient received mycophenolate mofetil, two patients received infliximab, and four patients received adalimumab. Both patients previously administered infliximab exhibited ADA formation (median duration of therapy: 12 months). Furthermore, adalimumab ADA were identified in seven of the eight patients (median duration of therapy: 13.5 months). One patient exhibited an inadequate response to adalimumab despite weekly dosing. Alongside tocilizumab therapy, four patients also received low-dose prednisolone, and two patients received methotrexate. However, these drugs had previously been prescribed as part of concomitant medication. Detailed medication information and observed side effects are presented in Table 1. The mean follow-up duration was 33 months (range: 12–36 months).

### Fluorescein angiography

**Fig. 4:**
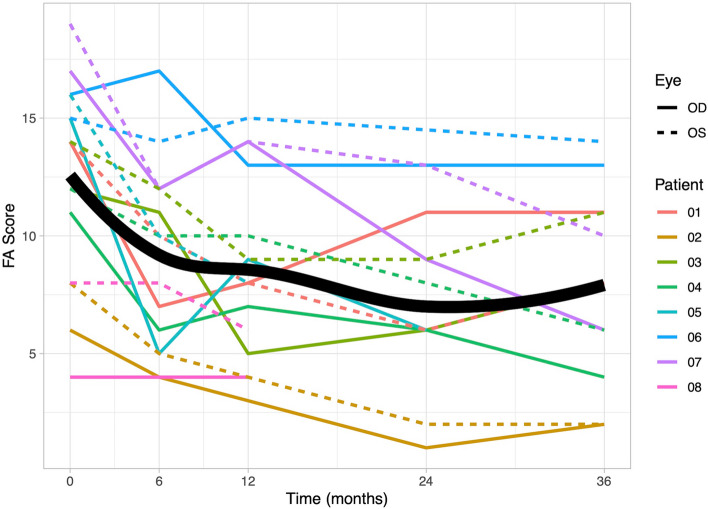
Graphical representation of fluorescein angiography (FA) scores. Individual patient data for FA scores are depicted over a 36-month observation period. At baseline, the recorded median FA score was 14 (quartiles: 10.25, 15.25), and it significantly decreased to 10 (5.75, 12) at 6-month. The FA score showed a diminished effect throughout the observation period. The scores for the right eyes are represented by solid lines, while the scores for the left eyes are represented by dashed lines. The bold line on the graph indicates the mean value of the scores, providing an overview of the central trend of the data over time

At baseline, the median recorded FA score was 14 (quartiles: 10.25, 15.25). Throughout the TCZ therapy, a significant linear decrease was observed (b = −0.14, *p* < 0.001; at 6 months: 10 (5.75, 12); at 36 months: 8 (5.5, 11)). The decrease showed a diminishing effect over time, establishing a significant quadratic relationship (*p* = 0.004). A detailed overview can be found in Fig. 4.

### Indocyanine green angiography

**Fig. 5:**
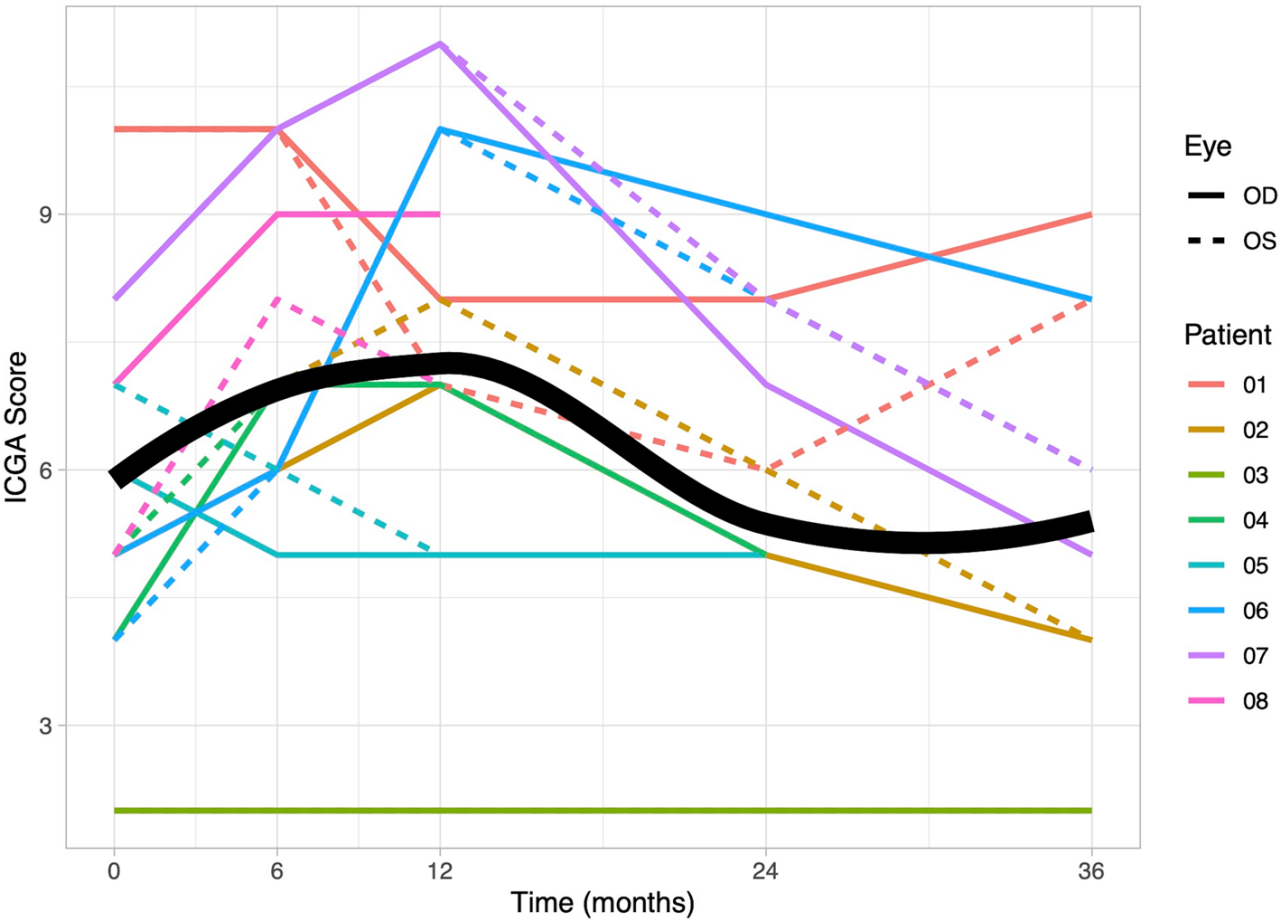
Graphical representation of indocyanine green angiography (ICGA) scores. Individual patient data for ICGA scores are depicted over a 36-month observation period. At baseline, the median ICGA score was recorded as 5 (quartiles: 4.75, 7.25). Over the first year of treatment, there was a significant increase in the mean ICGA score, reaching 7 (6, 9.25) at 6 months and 7 (6.5, 9.25) at 12 months. Following two years, the mean ICGA score returned to 5.5 (4, 7.5). The scores for the right eyes are represented by solid lines, while the scores for the left eyes are represented by dashed lines. The bold line on the graph indicates the mean value of the scores, providing an overview of the central trend of the data over time

In contrast to retinal inflammation, the ICGA score displayed an initial increase in choroidal inflammation during the first year of TCZ therapy (median at baseline: 5 (quartiles: 4.75, 7.25); at 6 months: 7 (6, 9.25); at 12 months: 7 (6.5, 9.25); *p* = 0.002). After two years, the ICGA score reverted to baseline levels (median at 24 months: 5 (5, 6.5); at 36 months: 5.5 (4, 7.5)), as depicted in Fig. 5. No significant improvement was observed over the entire observation period (b = −0.03, *p* = 0.13), while a significant quadratic effect indicated substantial increase followed by decrease until 24 months (*p* = 0.02).

### Vitreous haze

**Fig. 6:**
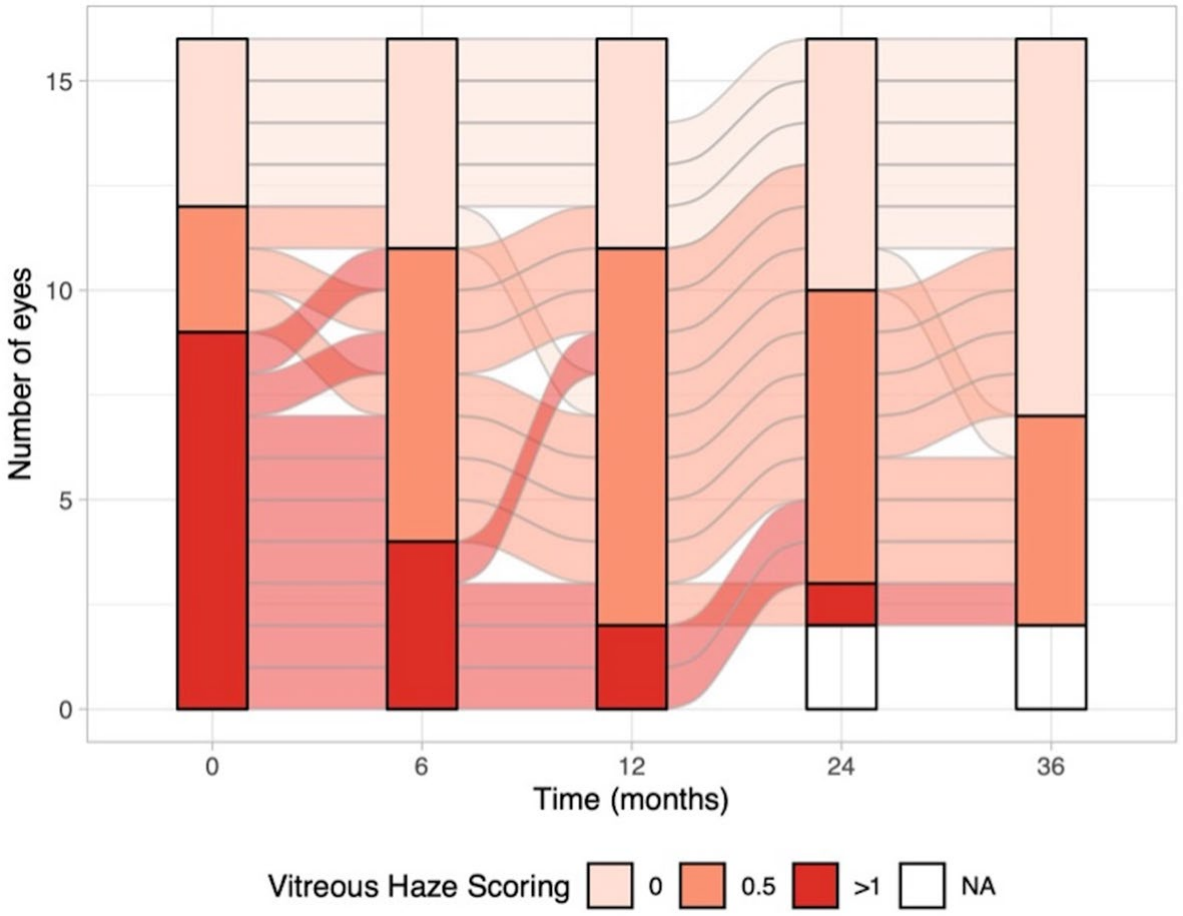
This figure illustrated the changes in Vitreous haze (VH) observed in individual eyes over a 36-month observation period. VH exhibited a significant decrease during the course of therapy (*p* = 0.02). At baseline, 9 out of 16 eyes had VH > 0.5. After 12 months of therapy, 2 out of 16 eyes had VH > 0.5, and at 36 months, 0 out of 14 eyes had VH > 0.5

VH decreased significantly during therapy (*p* = 0.02, at baseline: VH > 0.5 9/16 eyes (median: 1); at 12 months VH > 0.5 2/16 eyes (median: 0.5); at 36 months VH > 0.5 0/14 eyes (median: 0)). Detailed information can be found in Fig. 6.

### Visual acuity

**Fig. 7:**
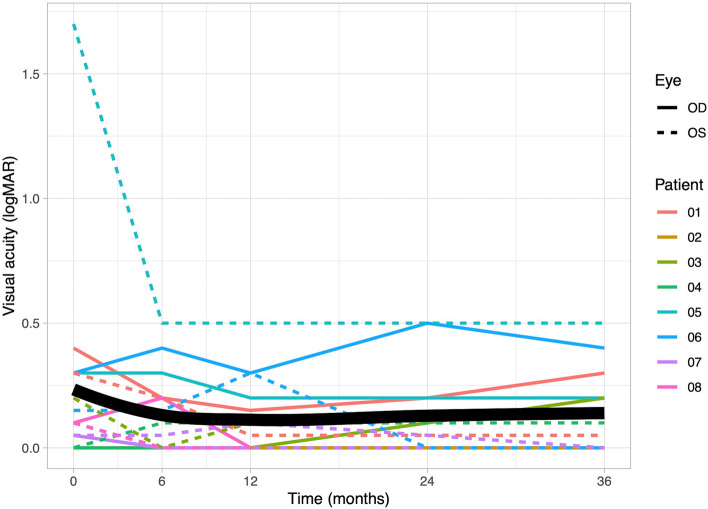
The figure presents the changes in Visual acuity (VA) measured in logarithm of minimum angle of resolution [logMAR] for individual patients over a 36-month observation period. VA exhibited slight improvement during the therapy (*p* = 0.31). The maximum improvement in VA was noted after 12 months of therapy. The VA for the right eyes is represented by solid lines, while the left eyes VA is represented by dashed lines. The graph’s bold line represents the mean value of VA

VA exhibited slight improvement during TCZ therapy (b = −0.01, *p* = 0.31). Compared to baseline, there was an increase in VA after 6 months (median at baseline: 0.10 logMAR (quartiles: 0.04, 0.30); at 6 months: 0.08 logMAR (0.00, 0.20)). Subsequently, the VA remained stable. The VA development is shown in Fig. 7.

### Central retinal thickness

**Fig. 8:**
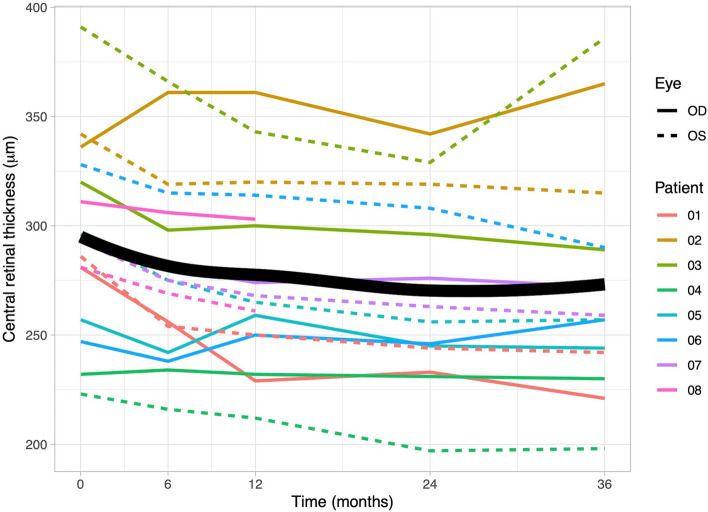
Changes in central retinal thickness (CRT) over the 36-month observation period. The figure illustrates the variation in CRT measured in micrometers [µm] for individual patients during 36-month observation period. At baseline, the mean CRT was 295 µm (quartiles: 275, 322), and it significantly declined over time (b = −0.58, *p* < 0.001). The lowest CRT level was reached after 36 months at 258 µm (243, 290). Solid lines represent data from right eyes, dashed lines from left eyes, and the bold line on the graph represents the mean CRT

The mean CRT at baseline was 295 µm (quartiles: 275, 322) (Fig. 8). Minor ME was observed in 13 out of 16 eyes. After 6 months of TCZ therapy, there was a significant decline in CRT, with a mean thickness of 275 µm (251, 308; *p* = 0.01). Subsequently, after 36 months, the minimum CRT level was reached (258 µm (243, 290)). Minor ME was present in 7 out of 12 eyes. A longitudinal analysis over the complete observation period revealed a significant decline (b = −0.58, *p* < 0.001).

## Discussion

The primary objective in uveitis management is to achieve remission, prevent complications, and deter recurrences. The long-term complications associated with corticosteroid treatment and the ineffectiveness of cDMARD or adalimumab, highlight the necessity for the use of other immunosuppressants.

TCZ, a recombinant monoclonal antibody targeting the IL-6 receptor, is currently utilized off-label for treating patients with refractory NIU. In this retrospective study, we aimed to evaluate the efficacy of TCZ in refractory BU over an extended duration. While previous long-term studies have frequently focused on ME [12, 15], our study addresses the long-term outcomes of inflammatory activity using FA and ICGA during TCZ therapy. Our findings provide valuable insights into the effectiveness of TCZ in BU and shed light on important considerations for its optimal use.

Longer experience has been gathered in the treatment of NIU using TCZ, particularly in patients with JIA and associated uveitis, owing to the drug´s approval for the joint disease. Calvo-Río et al. reported on 25 patients with JIA-associated refractory uveitis, demonstrating improvement in anterior segment inflammation in 79.2% of patients after six months of intravenous TCZ therapy (8 mg/kg every four weeks). They further demonstrated a notable increase in VA, a reduction in ME, and decreased dependence on additional steroid therapy [20]. Similarly, Maleki et al. observed eight JIA patients undergoing TCZ therapy for an average of 24.6 months (range: 9–70 months). The authors reported uveitis remission in five patients and a reduction of vasculitis after eight months in seven patients [21].

Evidence of uveitis improvement during TCZ therapy has been demonstrated in various diseases. In a study of 30 Behçet's disease patients, 67% achieved complete control of uveitis after six months [22]. Atienza-Mateo further observed a complete reduction of retinal vasculitis (*n* = 8) and choroiditis (*n* = 3) in Behçet's disease patients following an average of 9.5 months [23]. Moreover, TCZ has shown promise in addressing BU. A case report described two patients who experienced no recurrence of ME and a decrease in vitritis after 10 and 18 months of treatment [24].

Our study also demonstrated improvement in retinal vasculitis and choroidal inflammation in BU; however, a more precise description of these gradations could be achieved by implementing the ASUWOG score in clinical routine [17]. While FA and ICGA scores are not routinely assessed as disease markers in most clinical trials, they play a crucial role in evaluating retinal and choroidal inflammation. The FA and ICGA scoring system by ASUWOG group is a reliable method for assessing the degree of ocular inflammation within the retinal and choroidal compartments. This grading system complements the VH, which is the most commonly used parameter according to the SUN classification.

In this cohort of BU patients, TCZ therapy resulted in a significant improvement in retinal inflammatory activity, as evidenced by a decrease in FA scores over time. This effect was measurable after only six months and remained consistent throughout the observation period; a similar effect was observed for the VH score. Karaca et al. also employed the FA score, demonstrating an improvement in non-infectious retinal vasculitis in eight patients from 11.6 ± 4.4 to 5.8 ± 3.9 after six months [25]. In this study, a reduction over the same period was observed from 14 (quartiles: 10.25, 15.25) to 10 (5.75, 12), and after 36 months, it decreased further to 8 (5.5, 11). Wennink et al. similarly utilized ASUWOG classification and demonstrated a reduction in FA scores from a baseline of 14 to 8 after six months in seven children with non-anterior uveitis [26]. Two other studies reported a reduction in FA scores in NIU patients: after three months from 12.5 ± 4.3 to 5.1 ± 3.4 and after six months from 6.00 ± 3.26 to 2.00 ± 3.40 [14, 27]. These studies demonstrated a more profound reduction compared to our study, potentially owing to the intravenous application form in all these studies. However, none of the studies demonstrated a prolonged efficacy over the time course of 36 months.

Interestingly, the response of the choroidal inflammation might differ from that of the retinal vasculitis in response towards TCZ therapy. ICGA scores showed an initial increase within the first year of TCZ treatment, with a decline observed after 24- and 36-months. These findings suggest a delayed onset of the choroidal response to TCZ or potentially a limited effect on managing choroidal inflammation. The persistence of dark dots on ICGA in BU patients supports this observation. In some BU patients, the number of dark dots remained stable despite treatment, suggesting that these dots may not fully resolve with therapy once a certain degree of choroidal damage has occurred. However, other patients showed a reduction in the number of dark dots over time, potentially reflecting a delayed but positive response to TCZ. This variability highlights the challenges of relying on hypofluorescent spots as a definitive marker for resolving choroidal inflammation [28]. In this respect, underlying pathophysiology may provide insights. The effect of IL-6, which is inhibited by TCZ, is a breaking down of the blood-retinal barrier and promotion of retinal inflammation. As TCZ modifies the immune response, vascular remodeling in the choroid might take longer to manifest [29]. Further investigations are needed to understand the mechanisms behind this potentially differential response. Alternatively, new therapeutic options such as Janus kinase (JAK) inhibitors may potentially be more effective in managing the choroidal inflammation. Tao et al. reported successful treatment of two patients with Behçet's disease and panuveitis using Upadacitinib [30].

Primarily, TCZ has gained attention for its positive impact on reducing inflammatory ME [13, 15, 16, 31]. Our study similarly observed a reduction in CRT. However, at the beginning of TCZ therapy all patients presented with either normal CRT or only slight thickening and chronic intraretinal cysts. On the one hand, this can be explained by the chronic inflammation; on the other hand, it is important to note that six patients had previously received intravitreal dexamethasone implant to treat ME before starting TCZ therapy. Nevertheless, only one patient required additional dexamethasone re-inserting during TCZ therapy. Comparable to the data of Mesquida et al.´s findings, who observed a CRT reduction from 516 μm ± 55 at baseline to 274 μm ± 14 at 24 months, [15] our study demonstrates a significant CRT reduction from 295 µm (quartiles: 275, 322) at baseline to 258 µm (243, 290) at 36 months.

This study further observed a slight improvement in VA, from 0.10 logMAR (0.04, 0.30) at baseline to 0.08 logMAR (0.00, 0.20) after 6 months, indicating an overall good VA. In comparison, Mesquida et al. reported a VA gain from 0.78 ± 0.18 to 0.40 ± 0.17 after 24 months [15]. At baseline, one patient had low vision (≥ 1.0 logMAR) in one eye, and after six months, neither eye did not reach the threshold. Leclercq et al. reported 29.1% low vision patients, which decreased to 17% after six months of therapy [13]. However, our long-term results showed a slight decrease in VA after three years of therapy. These findings may indicate a diminished efficacy after a certain period of TCZ therapy. Similar loss of efficacy has been described for other biologic agents, potentially due to the development of ADA. In rheumatoid arthritis, the occurrence of ADA against TCZ has been recorded in 1.5% of patients and is significantly lower as compared to adalimumab or rituximab [32, 33]. This study did not investigate ADA against TCZ.

While the majority of studies focused on intravenous administration of TCZ, our study additionally reported subcutaneous administration, which is particularly convenient for patients. Randomized trials comparing intravenous versus subcutaneous administration in ophthalmology are not available. However, there are case reports documenting exacerbated inflammation after switching from intravenous to subcutaneous administration in patients with JIA uveitis and non-anterior NIU [34, 35]. Conversely, an analysis of the STOP trial revealed no significant difference in the efficacy of the standard 8 mg/kg compared to a reduced 4 mg/kg dose in improving or stabilizing NIU.[36] Additionally, Burlo et al. reported successful subcutaneous TCZ therapy in four out of five patients with NIU, showcasing comparable TCZ serum concentrations relative to intravenous administration [37]. Leclercq et al. demonstrated that there was no significant difference in the reduction of uveitic ME between intravenous (*n* = 39) and subcutaneous (*n* = 16) administration [13]. However, in the course of our study, three patients required intermittent switching to intravenous therapy due to inadequate inflammatory control with subcutaneous administration.

The safety of TCZ in patients has been assessed in various phase 3 and 4 randomized control trials [7, 38–40]. In our study, two patients experienced side effects (upper respiratory tract infections and exanthema), but there was no discontinuation of therapy. Upper respiratory tract infections were also reported as an adverse event in the STOP study, and two of the 37 patients experienced a low absolute neutrophil count, leading to the discontinuation of therapy in one patient.[16] Leclercq et al. observed adverse events in 12.7% of patients (with 54.6% being infections) and serious events in 5.5% during TCZ therapy (such as pneumonia, deep abscess, and injection-site reaction). However, it is not reported whether adverse events differed between the intravenous and subcutaneous administration [13].

While the study supports the effectiveness of TCZ in managing BU, it has certain limitations. The retrospective study design resulted in inconsistent observation times and incomplete performances of angiography. To minimize the influence of case numbers on the outcomes, we utilized longitudinal linear mixed models in the statistical analysis. Additionally, the calculation of the ASUWOG score is semiquantitative and relies on the investigator, which may introduce some subjectivity. The study includes relatively small number of patients, with both patient eyes included, which may impact the overall representation of results. Another limitation is the uncertain significance of immunosuppression in combination with tocilizumab therapy. In order to negate the impact of alternative immunosuppression, patients were selected based on exclusion criteria. Nevertheless, it is not possible to determine the extent to which the data may have been influenced by the long-lasting effects of intravitreal implants or the previous immunosuppression. Future research needs to perform prospective analyses to disentangle the effects of TCZ from those of other medications and to examine the individual effects of TCZ on outcomes of interest.

## Conclusion

In conclusion, our study demonstrates the long-term effect of TCZ on retinal and choroidal inflammation in refractory BU. These results highlight the efficacy of TCZ in reducing retinal vasculitis and enhancing visual outcomes over an extended follow-up period. However, our observations suggest that TCZ might be comparatively less effective in managing choroidal inflammation. Future prospective studies with larger sample sizes and the inclusion of control groups are warranted to determine the optimal treatment strategies for TCZ therapy in NIU.

## Data Availability

The datasets used and analyzed during the current study are available from the corresponding author on reasonable request.
